# Association between *TCF7L2* Genotype and Glycemic Control in Diabetic Patients Treated with Gliclazide

**DOI:** 10.1155/2013/374858

**Published:** 2013-02-20

**Authors:** Martin Javorský, Eva Babjaková, Lucia Klimčáková, Zbynek Schroner, Jozef Židzik, Mária Štolfová, Ján Šalagovič, Ivan Tkáč

**Affiliations:** ^1^Department of Internal Medicine 4, Faculty of Medicine, L. Pasteur University Hospital, P. J. Šafárik University in Košice, 041 90 Košice, Slovakia; ^2^Department of Medical Biology, Faculty of Medicine, P. J. Šafárik University in Košice, 040 66 Košice, Slovakia

## Abstract

Previous studies showed associations between variants in *TCF7L2* gene and the therapeutic response to sulfonylureas. All sulfonylureas stimulate insulin secretion by the closure of ATP-sensitive potassium (K_ATP_) channel. The aim of the present study was to compare *TCF7L2* genotype specific effect of gliclazide binding to K_ATP_ channel A-site (Group 1) with sulfonylureas binding to AB-site (Group 2). A total of 101 patients were treated with sulfonylureas for 6 months as an add-on therapy to the previous metformin treatment. *TCF7L2* rs7903146 C/T genotype was identified by real-time PCR with subsequent melting curve analysis. Analyses using the dominant genetic model showed significantly higher effect of gliclazide in the CC genotype group in comparison with combined CT + TT genotype group (1.32 ± 0.15% versus 0.73 ± 0.11%, *P* ^adj^ = 0.005). No significant difference in ΔHbA1c between the patients with CC genotype and the T-allele carriers was observed in Group 2. In the multivariate analysis, only the *TCF7L2* genotype (*P* = 0.006) and the baseline HbA1c (*P* < 0.001) were significant predictors of ΔHbA1c. After introducing an interaction term between the *TCF7L2* genotype and the sulfonylurea type into multivariate model, the interaction became a significant predictor (*P* = 0.023) of ΔHbA1c. The results indicate significantly higher difference in ΔHbA1c among the *TCF7L2* genotypes in patients treated with gliclazide than in patients treated with glimepiride, glibenclamide, or glipizide.

## 1. Introduction

Sulfonylureas belong to the most prescribed oral antidiabetic drugs worldwide. They act by the closure of K_ATP_ channel in pancreatic *β*-cells which results in membrane depolarization, calcium influx in *β*-cells, and subsequent insulin release [1]. K_ATP_ channel is composed of four pore forming potassium inward rectifier 6.2 (Kir6.2) subunits encoded by *KCNJ11* gene. The external part of the channel is constituted by four sulfonylurea receptor 1 (SUR1) subunits encoded by *ABCC8* gene [2]. Nonsynonymous variants *KCNJ11* E23K and *ABCC8* S1369A were identified which are in strong linkage disequilibrium [3]. Pharmacogenetic studies showed stronger effect of sulfonylureas, predominantly gliclazide, in the carriers of the genotypes *KCNJ11* K23 and/or *ABCC8* A1369 [4–6].

Single nucleotide polymorphisms (SNPs) of gene encoding transcription factor 7-like 2 (*TCF7L2*) were shown to have the strongest association with type 2 diabetes among all diabetes associated gene SNPs. The risk of developing diabetes is twice as high as that in homozygous carriers of the risk genotypes in comparison with homozygous carriers of common variants [7, 8]. Functional studies showed that *TCF7L2 *risk variants were associated with decreased insulin secretion [9, 10]. Pharmacogenetic studies reported a significant association between *TCF7L2* risk variants and lower effect of sulfonylurea treatment [11–13].

Gliclazide differs from the other commonly used sulfonylureas in several aspects. It binds exclusively on the A-site while the majority of other commonly used sulfonylureas bind to the AB-site of the K_ATP_ channel [15]. Recently, it was observed in a study on cell lines that K_ATP_ channel is more sensitive to inhibition by gliclazide, but not glimepiride, glibenclamide, or glipizide (all AB-site binding drugs) in the carriers of K23/A1369 risk haplotype in comparison with the carriers of E23/S1369 haplotype [4]. 

We hypothesized that a difference might exist also in *TCF7L2 *genotype effect on glucose reduction between gliclazide and the AB-site binding sulfonylureas. The aim of the present study was to compare genotype effect on the HbA1c reduction in the group of patients treated with gliclazide with the group of patients who used AB-site binding sulfonylureas—glimepiride, glibenclamide, and glipizide.

## 2. Methods

### 2.1. Patients and the Study Design

Type 2 diabetes was diagnosed according to the criteria of the American Diabetes Association [16]. The study was conducted in a university hospital setting. One hundred and one patients (50 males and 51 females) of Central European Caucasian origin were recruited from three outpatient clinics. Baseline clinical and biochemical characteristics of patients are shown in Table 1. Patients were eligible for the study if they were on previous metformin monotherapy for at least 6 months and failed to maintain HbA1c <7.0% on maximal tolerated doses of metformin at two consecutive visits within a three-month period. Inclusion criteria were HbA1c of 7.0%–11.0%, age 35–70 years, and body mass index (BMI) 20–35 kg/m^2^. Patients with malignancies, endocrine disorders, chronic renal failure, severe liver disease, systemic inflammatory disease, and corticosteroid treatment were excluded. The ethical approval for this study was obtained from the L. Pasteur University Hospital Review Board. All participating subjects gave a written consent to the study.

At the baseline visit, anthropometric data, as well as the diabetes duration and metformin treatment duration, were recorded. Blood samples were taken for genotyping and for biochemical measurements. Sulfonylurea treatment was started with 25%–50% of maximum approved dose for the specific sulfonylurea. A total 55 of patients were treated with gliclazide, and 46 patients were treated with the sulfonylureas binding to K_ATP_ channel AB-site: 29 patients with glimepiride, 14 patients with glibenclamide, and 3 patients with glipizide. The measurements of HbA1c were repeated after 3 and 6 months. If HbA1c level <7% was not reached after 3-month therapy, doses could have been increased up to 100% of the approved dose for the specific sulfonylurea compound. Mean sulfonylurea dose prescribed at the 3-month visit was 47 ± 2% of maximum approved dose for specific drug. Metformin dose was not changed during the entire study period. The participating physicians were blinded to the results of genotyping. The main study outcome was the difference between HbA1c level and baseline HbA1c (ΔHbA1c) following 6-month therapy with sulfonylurea.

### 2.2. Biochemical Methods and Genotyping

In all patients, peripheral venous blood samples were collected following an overnight fast. HbA1c was measured using an immunoturbidimetric method (Roche Diagnostics, France). Genomic DNA was extracted using a Wizard Genomic DNA purification kit (Promega Corp., Wisconsin, USA). PCR was performed in 10 *μ*L of reaction volume on LightScanner 32 instrument (Idaho Technology Inc., Salt Lake City, USA) at asymmetric primer ratio. Master mix comprised of 0.2x LCGreen Plus+ (Idaho Technology Inc.), 200 *μ*M dNTPs (Jena Bioscience, Jena, Germany), 0.05 *μ*M forward primer, 0.5 *μ*M reverse primer, 1 *μ*M unlabeled blocked probe, 3 mM MgCl_2_, 1U BioThermAB polymerase with 1x corresponding buffer (GeneCraft, Münster, Germany), and approximately 10 ng DNA. The sequences of oligonucleotides (Sigma-Aldrich, Germany) were the following:

 5′-CTCTGCCTCAAAACCTAGCACA-3′ (forward primer),
 5′-GTCTGAAAACTAAGGGTGCCTCAT-3′ (reverse primer),
 5′-GCACTTTTTAGATACTATATAATTTAATTGCC-3′phos (probe).

PCR conditions were the following: initial denaturation at 95°C for 5 min, 55 cycles at 95°C for 10 s, 64°C for 10 s, and 72°C for 10 s. Amplification was performed at the thermal transition rate of 10°C/s for all steps and was immediately followed by melting analysis with a denaturation at 95°C for 30 s and renaturation at 45°C for 1 minute. Data were acquired over 50–90°C range at the thermal transition rate of 0.1°C/s. Genotypes were identified by the melting temperatures of probe peaks on the normalized derivative plots using LightScanner 32 software 1.0.0.23 (Idaho Technology Inc.).

### 2.3. Statistical Analysis

Statistical analyses were performed using SPSS 17.0 for Windows software (SPSS Inc., Chicago, IL, USA). The continuous variables are presented as mean ± standard error of mean (SEM). For the comparison of continuous variables, unpaired/paired Student's *t*-test and analysis of variance (ANOVA) with post-hoc comparisons were used where appropriate. *χ* ^2^-test was used to test the Hardy-Weinberg equilibrium and for comparison of gender representation. Multivariate linear models were used for the testing of the response of HbA1c to sulfonylurea according to the genotypes. All models were adjusted for the age at the beginning of sulfonylurea treatment, gender, baseline BMI, baseline HbA1c, sulfonylurea type, and sulfonylurea dose which was standardized as a percentage of maximal doses for the specific sulfonylurea. 

## 3. Results

Anthropometric and biochemical characteristics of all study subjects and groups of patients treated either with gliclazide (Group 1) or with AB-site binding sulfonylureas (Group 2) are shown in Table 1. No significant difference was observed in gender representation, average age, BMI, diabetes duration, baseline HbA1c, HbA1c after 6 months, and sulfonylurea dose between the two groups. There was no significant difference between both groups in the average ΔHbA1c following 6-month therapy with sulfonylurea (Table 1). 

A total of 51 patients were homozygous for wild type C-allele (CC genotype), 41 patients were heterozygous (CT genotype), and 9 patients were homozygous for the type 2 diabetes associated T-allele (TT genotype) of *TCF7L2* rs7903146. Genotype distribution followed the Hardy-Weinberg equilibrium. Clinical characteristics of the study group according to the *TCF7L2 *genotypes are displayed in Table 2.

After 6 months of the sulfonylurea therapy, a significant difference among the genotypes in relation to ΔHbA1c was observed in both the entire study group and the gliclazide treated subgroup (Group 1), while no significant difference in effect among the genotypes was observed in Group 2 (Table 3). The biggest reduction in HbA1c was observed in CC genotype group, while the reductions were similar in both CT and TT genotype groups suggesting possible dominant way of inheritance (Table 3).

Further analyses using dominant genetic model showed significantly higher effect of gliclazide in the CC genotype group on HbA1c reduction in comparison with combined CT + TT genotype group (1.32 ± 0.15% versus 0.73 ± 0.11%, *P* = 0.003, *p* ^adj^ = 0.005). In contrast, no significant difference in ΔHbA1c between the patients with CC genotype and T-allele carriers was observed in Group 2 (Table 3). 

In the multiple linear regression model with ΔHbA1c as dependent variable, *TCF7L2* genotype, age, gender, BMI, baseline HbA1c, sulfonylurea group, and sulfonylurea dose were included as independent variables (Table 4). In this model the *TCF7L2* genotype (*P* = 0.006) and the baseline HbA1c (*P* < 0.001) were the only significant predictors of ΔHbA1c (*r* ^2^ = 0.56). After introducing the interaction term between *TCF7L2* genotype and sulfonylurea treatment group to the model, the variance explained by the model increased (*r* ^2^ = 0.58) and the interaction term became a significant predictor (*P* = 0.023) of ΔHbA1c (Table 4).

## 4. Discussion

The main finding of the present study is a significant interaction found between *TCF7L2* genotype and the type of sulfonylurea used in the treatment of the patients with type 2 diabetes. The patients treated with gliclazide had significantly stronger genotype specific effect with the average reduction in HbA1c in homozygous carriers of common C-allele higher by 80% than in-risk T-allele carriers. No significant genotype effect was observed in the group of patients treated by glibenclamide, glimepiride, or glipizide. 

To the best of our knowledge, only three studies analyzed the effect of sulfonylurea treatment in relation to *TCF7L2* genotype. Pearson et al. found higher probability of sulfonylurea failure and smaller reduction in HbA1c in *TCF7L2* rs1225372 and rs7903146 risk allele carriers in a group of 901 patients included in the Genetics of Diabetes Audit and Research Tayside study (GoDARTs) [11]. The results observed in GoDARTs were replicated independently by two Central European groups [12, 13]. In none of the mentioned studies, the results were analyzed according to used sulfonylurea type [11–13]. The present study extends the current knowledge by demonstrating the first observation of the different *TCF7L2* genotype effect of various sulfonylureas with the strongest genetic specificity observed in gliclazide users in contrast to the patients treated with other sulfonylurea drugs, as proved by the test of interaction. 

**Figure 1:**
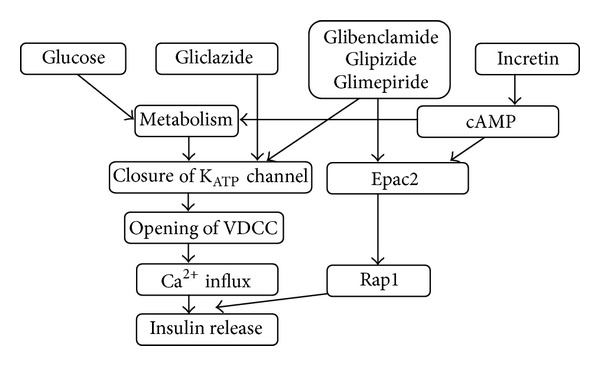
Convergence of the sulfonylurea and incretin pathways on insulin secretion via Epac2 [14]. cAMP: cyclic adenosine monophosphate; K_ATP_: ATP-dependent potassium channel; VDCC: voltage dependent calcium channel; Epac2: exchange protein activated by cAMP 2; Rap1: Ras-like guanosine phosphatase.

The explanation of this difference might lie in the different pharmacodynamic characteristics of gliclazide and the other studied sulfonylureas. Beside the mentioned K_ATP_ channel binding site specificity, there are further differences between gliclazide and other sulfonylureas. Some studies relate the *TCF7L2* effect to the action of incretin hormones—glucagon-like peptide 1 (GLP-1) and glucose-dependent insulinotropic peptide (GIP) [17]. These hormones stimulate *β* cells primarily by the activation of the cAMP-dependent pathway [18]. Interestingly, it was recently shown that beside their effect on closure of K_ATP_ channel, the majority of sulfonylureas also activate the exchange protein activated by cAMP 2 (Epac2) which subsequently activates small G-protein Rap1. Epac2/Rap1 signaling is essential for potentiating the first phase of insulin release [19]. While in studies in animals and cell lines tolbutamide, glibenclamide, chlorpropamide, and glipizide were able to activate Epac2/Rap1 signaling, gliclazide did not activate this pathway [14, 20]. Because the T-allele at *TCF7L2* rs7903146 has been shown to be related to incretin resistance [21], drugs that activate Epac2 such as glimepiride or glibenclamide may attenuate the deficit incurred by *TCF7L2* genotype, whereas a drug like gliclazide might be unable to do so (Figure 1). Whether the mentioned differences in the mechanism of action explain the pharmacogenetic difference between gliclazide and the other sulfonylureas is not clear. It is possible that unknown pathogenetic mechanisms may be involved, and further functional studies are required. 

The present study has some limitations. With respect to relatively small sample size, it had limited statistical power to detect small genotype-related differences. Because of its exploratory character, replications in independent study cohorts are needed. 

## 5. Conclusion

In the diabetic patients treated by gliclazide, we observed bigger reduction in HbA1c by 0.6% in approximately 50% of patients with the common CC genotype, in comparison with the risk *TCF7L2 *rs7903146 T-allele carriers. The magnitude of difference may have practical implications; for example, with the aim to overcome the genetic defect; the carriers of *TCF7L2* T-allele might need higher doses of gliclazide, a sulfonylurea drug with good evidence base and safety profile [22, 23].

## Figures and Tables

**Table 1:** Clinical and biochemical characteristics of the patients according to treatment with gliclazide (Group 1) or K_ATP_ channel AB-site binding sulfonylureas (Group 2).

	Entire group (*n* = 101)	Group 1 (*n* = 55)	Group 2 (*n* = 46)	*P*
Sex (males/females)	50/51	29/26	21/25	0.921
Age (years)	61.9 ± 1.0	62.3 ± 1.2	61.4 ± 1.7	0.673
Diabetes duration (years)	2.3 ± 0.2	2.3 ± 0.2	2.4 ± 0.5	0.839
Baseline BMI (kg/m^2^)	30.6 ± 0.40	30.7 ± 0.5	30.5 ± 0.6	0.729
SU dose (% max dose)	47.0 ± 1.9	44.5 ± 2.4	49.8 ± 2.9	0.160
Baseline HbA1c (%)	8.04 ± 0.09	7.91 ± 0.11	8.20 ± 0.15	0.122
HbA1c after 6 months (%)	6.99 ± 0.06	6.89 ± 0.09	7.11 ± 0.09	0.094
ΔHbA1c (%)	1.05 ± 0.08	1.03 ± 0.10	1.09 ± 0.11	0.717

BMI: body mass index; SU: sulfonylurea derivatives; *P* values for difference between Group 1 and Group 2.

**Table 2:** Baseline characteristics across *TCF7L2 *rs7903146 genotypes in the entire group.

Entire group (*n* = 101)	CC (*n* = 51)	CT (*n* = 41)	TT (*n* = 9)	*P*
Sex (males/females)	28/23	18/23	4/5	0.535
Age (years)	61.7 ± 1.4	62.0 ± 1.6	62.9 ± 3.8	0.945
Diabetes duration (years)	2.6 ± 0.4	2.0 ± 0.2	2.0 ± 0.8	0.444
Baseline BMI (kg/m^2^)	31.0 ± 0.7	30.1 ± 0.4	30.7 ± 0.9	0.601
Baseline HbA1c (%)	8.06 ± 0.14	8.01 ± 0.13	8.06 ± 0.27	0.954
SU dose (% max dose)	43.6 ± 2.3	50.6 ± 3.3	49.1 ± 5.5	0.195

*P* values for *χ* ^2^-test (gender) and for ANOVA.

**Table 3:** Effect of the different sulfonylurea derivatives on ΔHbA1c with respect to *TCF7L2 *genotypes.

Entire group (*n* = 101)	CC (*n* = 51)	CT (*n* = 41)	TT (*n* = 9)	*P*	*P* ^adj^	
ΔHbA1c (%)	1.23 ± 0.11	0.89 ± 0.09	0.85 ± 0.31	0.064	0.022^a^	
Dominant model	CC (*n* = 51)	CT + TT (*n* = 50)			
ΔHbA1c (%)	1.23 ± 0.11	0.88 ± 0.09		0.019	0.006	
Group 1 (*n* = 55)	CC (*n* = 28)	CT (*n* = 21)	TT (*n* = 6)		
ΔHbA1c (%)	1.32 ± 0.15	0.76 ± 0.10	0.61 ± 0.40	0.010	0.013^b^	
Dominant model	CC (*n* = 28)	CT + TT (*n* = 27)			
ΔHbA1c (%)	1.32 ± 0.15	0.73 ± 0.11		0.003	0.005	
Group 2 (*n* = 46)	CC (*n* = 23)	CT (*n* = 20)	TT (*n* = 3)		
ΔHbA1c (%)	1.12 ± 0.18	1.01 ± 0.16	1.33 ± 0.39	0.775	0.810	
Dominant model	CC (*n* = 23)	CT + TT (*n* = 23)			
ΔHbA1c (%)	1.12 ± 0.18	1.06 ± 0.14		0.792	0.783	

*P* value for ANOVA, *P* ^adj^ value adjusted in general linear models for age, gender, baseline HbA1c, baseline BMI, and sulfonylurea dose. Post hoc comparisons between genotype groups: ^a^CC versus CT, *P* = 0.014, CC versus TT, *P* = 0.06; ^b^CC versus CT, *P* = 0.022, CC versus TT, *P* = 0.013.

**Table 4:** Multivariate predictors of ΔHbA1c after sulfonylurea treatment.

Independent variables	Model 1	Model 2
	*P*	*P*
*TCF7L2* genotype	0.006	0.002
Sulfonylurea type	0.419	0.028
*TCF7L2 *genotype ∗ sulfonylurea type	—	0.023
HbA1c baseline	<0.001	<0.001
Age	0.801	0.713
Gender	0.385	0.335
BMI	0.246	0.240
Sulfonylurea dose	0.218	0.380

Coding of the variables: *TCF7L2 *genotype: CC-0, CT + TT-1; sulfonylurea type: gliclazide-1, other sulfonylureas-2; gender: male-1, female-2.
